# Ultrastructural Observation and Gene Expression Profiling of *Schistosoma japonicum* Derived from Two Natural Reservoir Hosts, Water Buffalo and Yellow Cattle

**DOI:** 10.1371/journal.pone.0047660

**Published:** 2012-10-26

**Authors:** Jianmei Yang, Xingang Feng, Zhiqiang Fu, Chunxiu Yuan, Yang Hong, Yaojun Shi, Min Zhang, Jinming Liu, Hao Li, Ke Lu, Jiaojiao Lin

**Affiliations:** Shanghai Veterinary Research Institute, Chinese Academy of Agricultural Sciences, Key Laboratory of Animal Parasitology, Ministry of Agriculture of China, Shanghai, People’s Republic of China; National Institute of Environmental and Health Sciences, United States of America

## Abstract

Water buffalo and yellow cattle are the two of the most important natural reservoir hosts for *Schistosoma japonicum* in endemic areas of China, although their susceptibility differs, with water buffalo being less conducive to the growth and development of *S. japonicum.* Results from the current study show that the general morphology and ultrastructure of adult schistosomes derived from the two hosts also differed. Using high-throughput microarray technology, we also compared the gene expression profiles of adult schistosomes derived from the two hosts. We identified genes that were differentially expressed in worms from the two natural hosts. Further analysis revealed that genes associated with protein kinase and phosphatase, the stimulus response, and lipid and nucleotide metabolism were overexpressed, whereas genes associated with reproduction, anatomical structure morphogenesis and multifunctional motif were underexpressed in schistosomes from water buffalo. These differentially expressed genes were mainly involved in nucleotide, energy, lipid metabolism, energy metabolism, transcription, transport and signaling pathway. This suggests that they are key molecules affecting the survival and development of schistosomes in different natural host species. The results of this study add to current understanding of the interplay between parasites and their natural hosts, and provide valuable information for the screening of vaccine candidates or new drug targets against schistosomiasis in the natural reservoir hosts in endemic areas.

## Introduction

Schistosomiasis is one of the most prevalent zoonotic diseases in the world, affecting approximately 250 million people and posing a risk to another 600 million [1]. Schistosomiasis control in China has been remarkably successful, with the number of cases being reduced from 11 000 000 to 326 000 by the end of 2010 [2]. Endemic areas of uncontrolled schistosomiasis are mostly distributed in the marsh, lake and mountainous regions of China [3]. Epidemiology surveys have shown that domestic animals also have an important role in the transmission of schistosomiasis in these areas [4]. Water buffalo and yellow cattle are the two major domestic animals reared in schistosomiasis-endemic areas of China as they are able to spread more eggs into the environment compared with human and other animal hosts. They are considered to be the main transmission source (i.e. reservoir hosts) for schistosomiasis in China and, therefore, should have an important role for prevention strategies [5], [6]. In endemic areas, control measures, including chemotherapy for bovine hosts, have both improved the health status of animals and reduced the spread of schistosomiasis in humans; however, the need for chemotherapy is ongoing and costly in terms of material resources and human input. Therefore, the development of an effective vaccine for animals and/or humans is a more sustainable choice for the control of this debilitating disease [7].

*Schistosoma japonicum* has a wide range of host species, with at least 46 species of mammal, other than humans, known to be naturally infected by *S. japonicum*, including a variety of domestic and wild animals, although only some are a schistosomiasis threat to humans [8]. Previous studies revealed the variability in the susceptibility of different types of host to *S. japonicum* infection, with yellow cattle, goats, mice and rabbits being more susceptible for infection compared with water buffalo, rats, pigs and horses [9]. In addition, the parasite clearance phenomenon has been observed in water buffalo after a certain period of infection [10]. Parasites that survive in this host also showed substantial changes in morphology, being shorter in length, having poorly developed gonads, demonstrating a lower rate of worm pairing and also of spawning by female worms [11], [12]. However, the factors that determine such differences in *S. japonicum* infection among natural reservoir hosts remain to be clarified.

Recent studies in our laboratory revealed that schistosomula of *S. japonicum* from susceptible host BALB/c mice, less susceptible host Wistar rat and the non-permissive host *Microtus fortis* displayed different expression profiles at the transcript and protein level [13], [14]. The studies suggested that investigating the gene or protein expression difference in worms from different hosts could provide useful information for understanding the mechanism determining the survival, development and maturing of schistosomes in their hosts. In addition, the studies also suggested that the gene or protein expression profiles in schistosomes from natural hosts would be different with those from laboratory animals. Therefore, to better elucidate the developmental mechanism of schistosomes in their natural hosts, as well as to identify the molecules that might affecting schistosome development, we infected water buffalo and yellow cattle with *S. japonicum*, and analyzed and compared the gene expression profiles of the parasites from the two natural hosts using a microarray technique. Our results are likely to be helpful for screening vaccine candidates or new drug targets for the control of schistosomiasis in the natural reservoir hosts in endemic areas.

**Figure 1:**
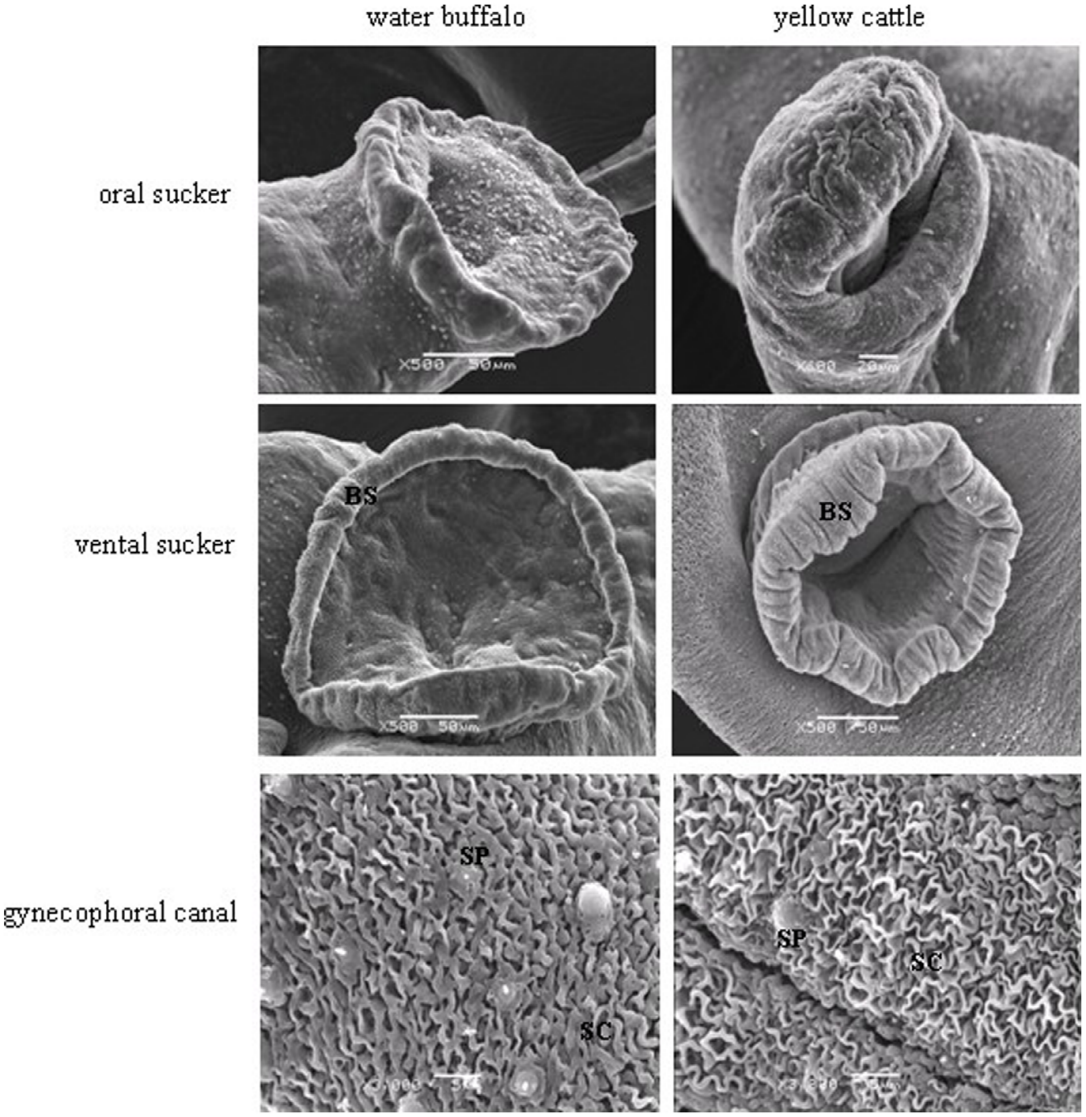
Scanning electron micrographs of the oral sucker, ventral sucker and the posterior segment of the gynecophoral canal of male worms derived from water buffalo and from yellow cattle. BS, border spine; SC, surface crest; SP, sensory papillae.

**Figure 2:**
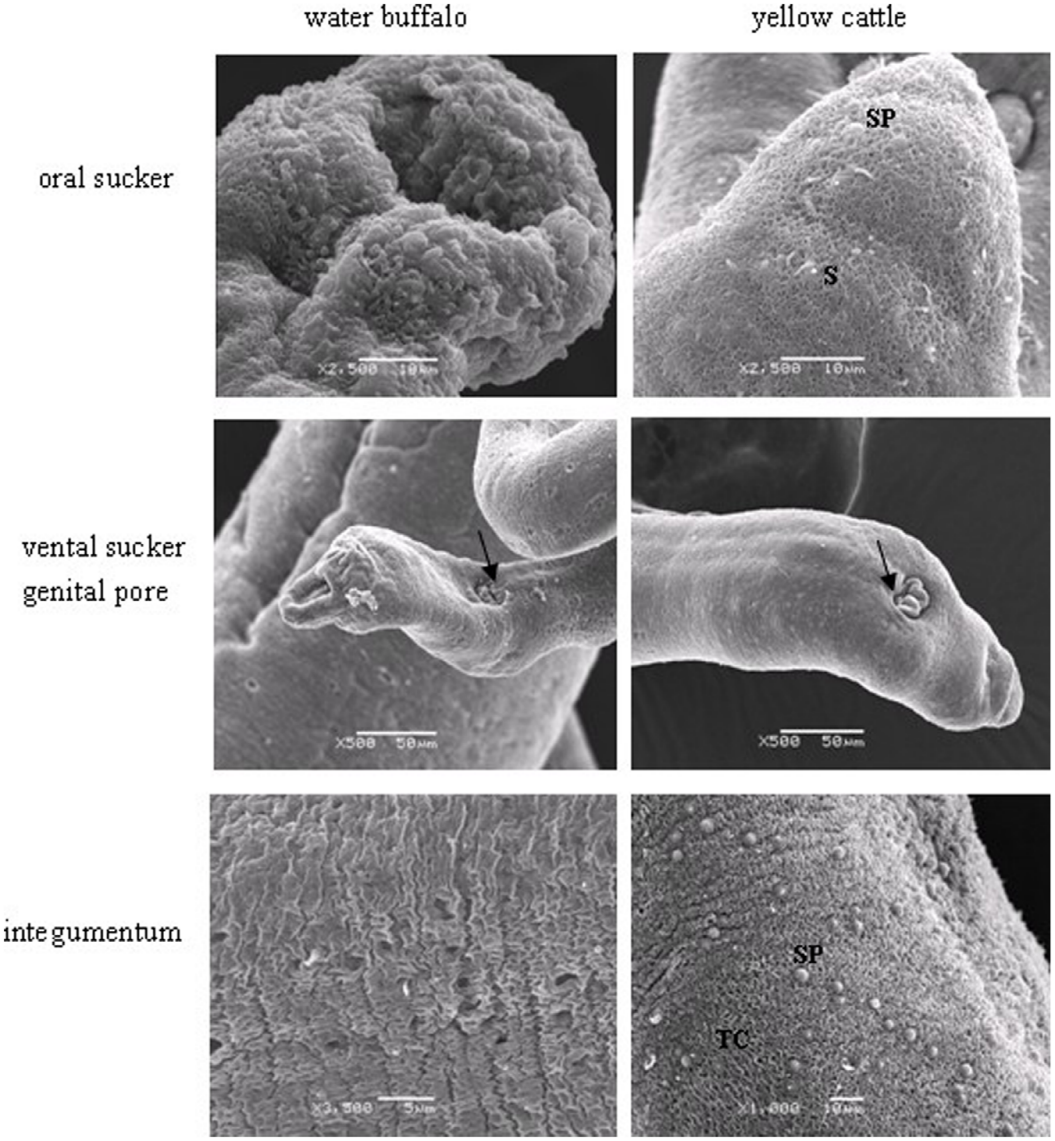
Scanning electron micrographs of the oral sucker, ventral sucker, genital pore (arrowheads) and the middle of the integumentum of female worms derived from water buffalo and yellow cattle. S, spine; SP, sensory papillae; TC, tegumental crest.

## Materials and Methods

### Animals and Infection

Three male water buffalo and three male yellow cattle, all 15–18 months old, with a similar body weight, and free of other parasitic helminth infection and other infectious diseases, were purchased from schistosome non-endemic areas and used for experimental infection. All animals were housed in covered pens, cared for by trained animal keepers and fed with hay and a commercial pelleted ration. *Schistosoma japonicum* (Chinese mainland strain) cercariae were obtained from the snail-maintaining room at Shanghai Veterinary Research Institute, Chinese Academy of Agricultural Sciences (CAAS). The water buffalo and yellow cattle were challenged percutaneously with the cecariae through the upper back using the cover glass method [15]. The study protocol was approved by the Animal Care and Use Committee of the Shanghai Veterinary Research Institute, CAAS.

**Figure 3:**
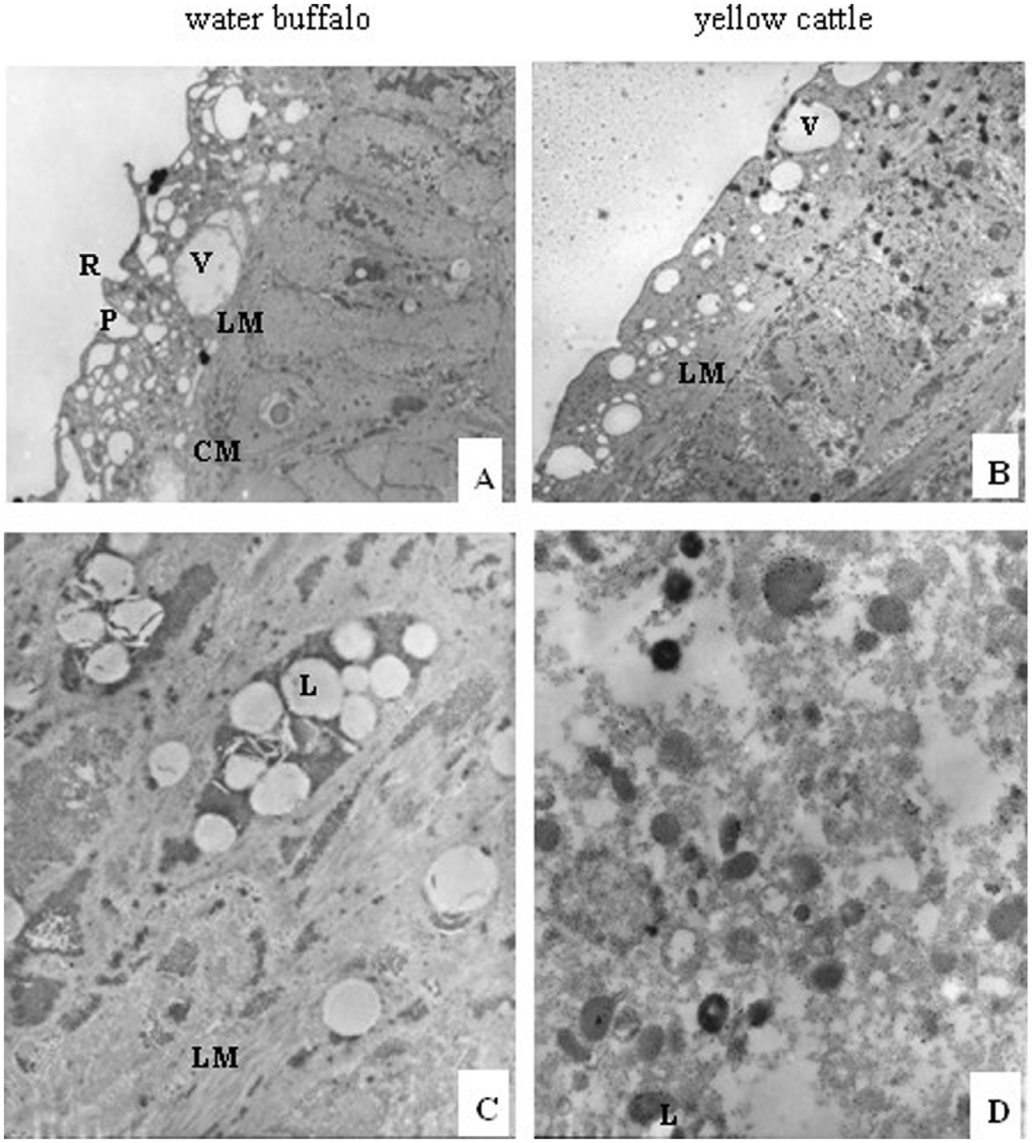
Transmission electron micrographs of male schistosomes derived from water buffalo and yellow cattle. (A,B) tegument, 2000×; (C,D) subtegument and inner structure, 12000×. CM, circular muscle; L, lipid droplet; LM, longitudinal muscle; P, pore; R, ridge; V, vacuole.

**Figure 4:**
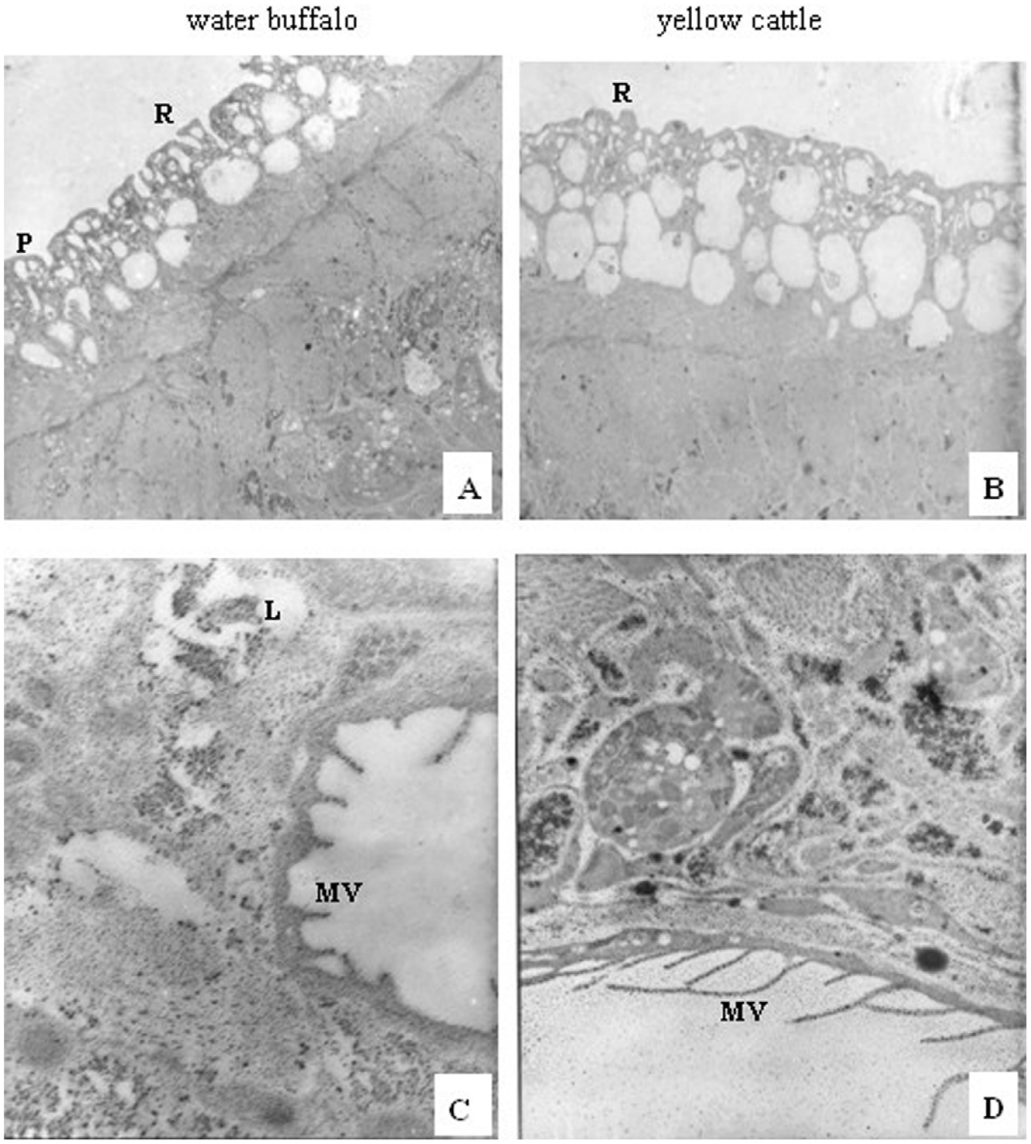
Transmission electron micrographs of female schistosomes derived from water buffalo and yellow cattle. (A,B) tegument, 2000×; (C,D) subtegument and inner structure, 12000×. L, lipid droplet; MV, microvilli; P, pore; R, ridge.

### Worm Collection and Ultrastructural Observation

The animals were sacrificed 7 weeks post-infection and the parasites were perfused through the hepatic portal vein. The male and female worms were detached manually, counted and their lengths and widths were measured by the same investigator. Worm samples were collected and stored in RNAlater (Amibion). The remaining worms were fixed in 2.5% glutaraldehyde phosphate buffer solution after washing with PBS (pH 7.4) three times, with a 15 min interval, and then fixed for 1.5 h with 1% osmic acid, and then washed three times as above, dehydrated with gradient alcohol (30%, 50%, 70%, 80%, 90%, 95% and 100%), vacuum dried and spurted for 3 min by an ion sputtering instrument. The samples were observed with JEOL (JSM-6380LV, Japan) by scanning electron microscopy (SEM). The samples were further dehydrated with acetone twice, embedded in an embedding medium, cut ultrathinly for a 70-nm section and then observed using an electron microscope (Hitachi H-600, Japan) by transmission electron microscopy (TEM).

**Figure 5:**
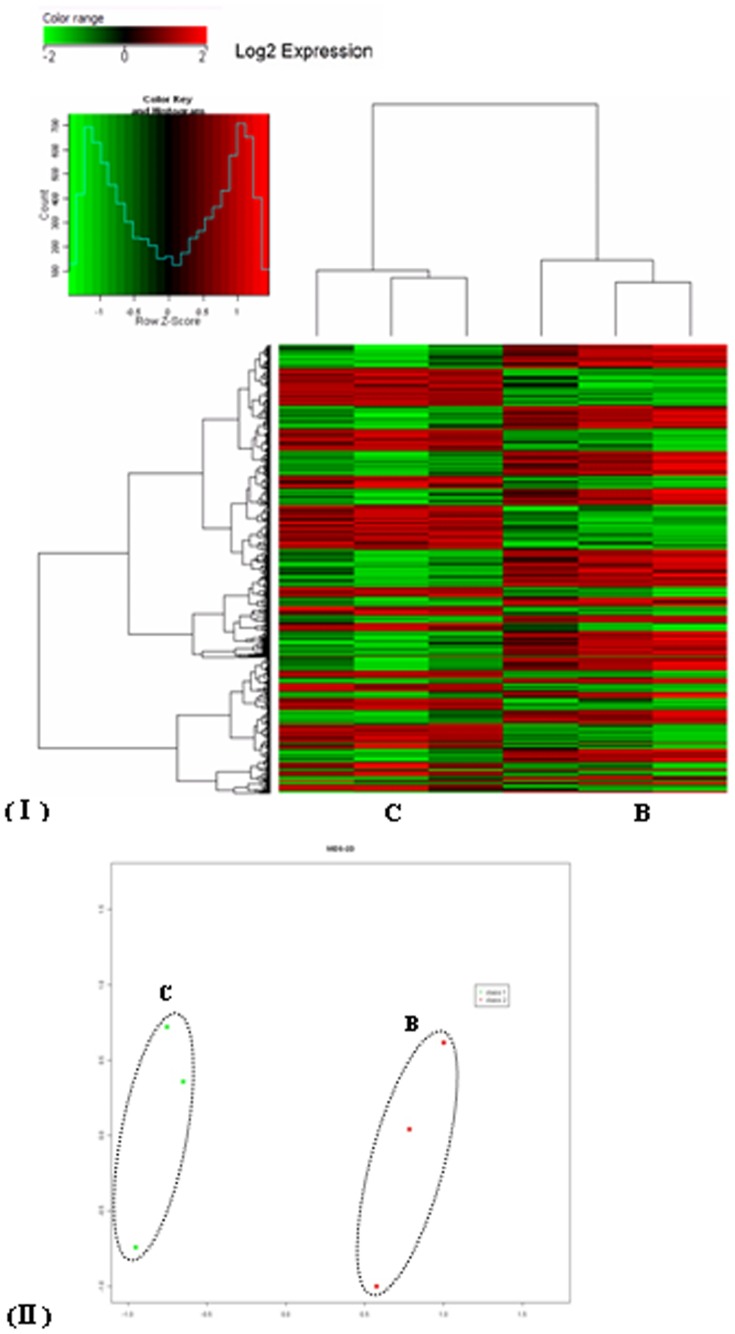
Transcription profile analysis of significant differentially expressed genes in *Schistosoma japonicum* from water buffalo (Group B) and yellow cattle (Group C). (I) Hierarchical clustering using genes (probe sets) showing differential expression (ANOVA *p*<0.05); (II) Principal component analysis of transcript profiles from Groups B and C.

### Microarray Composition

The microarray used to analyze gene expression in schistosomes from water buffalo and yellow cattle was constructed by Agilent Technologies (USA). The microarray design was based on sequence data for *S. japonicum*; 60-mer probes on the microarray included 13,821 contiguous sequences (contigs), plus proprietary positive and negative controls, including 12,645 from LSBI (http://lifecenter.sgst.cn/schistosoma/cn/genomeProject.do), 940 from EMBL (http://www.ebi.ac.uk/ebisearch/search.ebi?db=emblrelease_con&t=schistosoma), 48 from Genebank (http://www.ncbi.nlm.nih.gov/sites/entrez?db=nucleotide, 91 from http://www.chgc.sh.cn/japonicum/) and 37 from the *S. japonicum* cDNA library in our laboratory [16]. Full details of this schistosome microarray design have been deposited in the Gene Expression Omnibus (GEO) public database with the platform accession number GPL10987.

**Table 1:** Details of real-time RT-PCR primers and confirmation of results.

Probe name	Primer (5′-3′)	Size (bp)	Description	Microarray FC^a^	Real-time FC^b^
CUST_3643	FP: gcgcttctgggttaatgaag; RP: cagcaaacggtccatactg	207	Cathepsin L-like proteinase precursor(EC 3.4.22)	2.63	2.49
CUST_12988	FP: ctgctgtggagggaatgttt; RP: tggaggattccaggtttcag	219	Putative uncharacterized protein C14orf165	8.86	11.14
CUST_4112	FR:gttattggatttcccgctca; PR:atggcaatgaaagtgcatca	199	Cdk6, cyclin-dependent kinase 6 (EC:2.7.11.22, ko:K02091)	18.52	14.39
CUST_10350	FR: ggattgattccgccattac; PR:gaatggcagtattggttgacg	198	Asparagine-rich protein (Ag319) (ARP)(fragment)	6.11	5.87
CUST_4819	FP:ttaagcgggatcaatggaag; RP: caccacgacgtgttaattgc	210	SLC6A12, solute carrier family 6(neurotransmitter transporter, betaine/GABA), member 12 (ko:K05045)	−3.07	−2.2
CUST_10723	FR: tgtgccgttattgcgtttag; PR: attatcgcttttgccgtcag	178	Expressed protein	4.21	2.41
CUST_4101	FR:atactggtgagcggcctatg; PR:gcattcgcaaaccatgtaga	230	Iroquois-class homeodomain protein IRX-3 (Iroquois homeobox protein 3)	2.66	1.77
CUST_5523	FR: gcgttcgccaattgaattat; PR: tgttgtattgggtggggatt	184	Putative eukaryotic translation initiationfactor 3 subunit (eIF-3)	2.03	1.88
CUST_10057	FR:catgttcaatgggaagtgga; PR: tgcgctggttcaaaatgtaa	161	Protocadherin gamma B2 precursor	7.95	5.68

a Fold changes (FC) are expressed as the ratio of gene expression in schistosomes from water buffalo compared with schistosomes from yellow cattle; *p*<0.05, FDR <0.1.

b Mean -fold change in real-time PCR results for validation.

### RNA Extraction and Microarray Experiments

Total RNA was extracted from the parasites collected from water buffalo and yellow cattle using Trizol reagent (Invitrogen Life Technologies) and purified using RNAeasy mini kit (Qiagen) according to the manufacturer’s instructions. The RNA integrity and RNA quality was detected by the Agilent 2100 bioanalyzer (Agilent Technologies), the QC criteria was 2100RIN> = 7.0 and 28S/18S> = 0.7.

**Table 2:** Selected genes overexpressed in schistosomes from water buffalo compared with those from yellow cattle^a^.

Probe name	Accession	Gene and/or protein homology	Fold change	*p* value	FDR
**Protein kinase/phosphatase**					
CUST_4112	CNUS0000098407	Cyclin-dependent kinase 6	18.52	0.029	0.092
CUST_9981	CNUS0000104279	Serine/threonine-protein phosphatase 2A	4.54	6.51E-05	0
**Stimulus response associated with nervous system and transport**					
CUST_10057	CNUS0000104355	Protocadherin gamma B2 precursor	7.95	0.036	0.092
CUST_3217	CNUS0000097512	Metabotropic glutamate receptor 7 precursor	2.35	0.04	0.092
CUST_2092	CNUS0000096387	Regulating synaptic membrane exocytosis protein 2(Rab3-interacting molecule 2)	2.07	0.018	0.092
CUST_1940	CNUS0000096235	V-type H+-transporting ATPase subunit H	2.19	0.04	0.095
CUST_11483	CNUS0000105782	Ecotropic virus integration site 1 protein (EVI-1)	2.13	0.012	0.092
CUST_12289	CNUS0000106588	Equilibrative Nucleoside Transporter	2.01	0.035	0.092
**Lipid metabolism**					
CUST_5733	CNUS0000100029	Elongase of very long chain fatty acids protein 1	2.57	0.039	0.092
CUST_10029	CNUS0000104327	Elongase of very long chain fatty acids protein 4	2.15	0.052	0.092
CUST_12988	CNUS0000107293	Putative uncharacterized protein C14orf165, sphingosineN-acyltransferase;	8.86	0.007	0.073
**Nucleotide metabolism, transcription and translation**					
CUST_7791	CNUS0000102089	Nucleoside diphosphate kinase type 6	2.02	0.027	0.092
CUST_11457	CNUS0000105756	Histone H; nucleosome, DNA binding	2.36	0.006	0.092
CUST_2605	CNUS0000096900	Histone H4; nucleosome, DNA binding	2.27	0.009	0.092
CUST_8420	CNUS0000102718	Pumilio [S. mansoni]; RNA-binding	2.11	0.04	0.092
CUST_4101	CNUS0000098396	Iroquois homeobox protein 3, IRX-3; RNA polymerase II transcription factor activity	2.66	0.031	0.092
CUST_2965	CNUS0000097260	LIM/homeobox protein Lhx1; transcription regulationactivity	2.23	0.024	0.092
CUST_1782	CNUS0000096077	Eukaryotic translation initiation factor 3 subunit	5.33	0.009	0.092
CUST_7137	CNUS0000101434	39S ribosomal protein L35;ribosome composition,translation	2.08	0.02	0.092

a The list includes the probe name, gene accession, fold change (Fold), p value and q value the protein homology (i.e. the result of BlastX). In the gene accession line, the name such as CNUS0000098407 is from LSBI (http://lifecenter.sgst.cn/schistosoma/cn/genomeProject.do), the name such as FN326902 is from EMBL (http://www.ebi.ac.uk/embl/). FDR(q-value) is applied for false discovery rate control by R statistical language (http://www.bioconductor.org/packages/release/bioc/html/qvalue.html).

**Table 3:** Selected genes underexpressed in schistosomes from water buffalo compared with those from yellow cattle.

Probe name	Accession number	Gene and/or protein homology	Fold change	*p* value	FDR
**Reproduction**					
CUST_862	FN326855	Egg-secreted protein ESP15-like [Schistosoma mansoni]	–16.10	0.042	0.068
CUST_10799	CNUS0000105097	Imb-3 importin Beta family	–2.92	0.024	0.068
CUST_11761	CNUS0000106060	Homeobox protein distal-less dlx	–2.22	0.043	0.1
CUST_13255	CNUS0000107560	IPR000949 ELM2, domain-containing protein	–2.08	0.028	0.095
CUST_4819	CNUS0000099115	Taurine transporter, neurotransmitter transporter, ko:K05045	–2.07	0.006	0.068
**Anatomical structure morphogenesis**					
CUST_11761	CNUS0000106060	Homeobox protein distal-less dlx	–2.22	0.043	0.1
CUST_13255	CNUS0000107560	IPR000949 ELM2, domain-containing protein	–2.08	0.028	0.095
CUST_882	FN326902	Putative transmembrane protein 57 [Schistosoma japonicum]	–20.47	0.02	0.068
CUST_10799	CNUS0000105097	Imb-3 importin Beta family	–2.92	0.024	0.068
CUST_8162	CNUS0000102460	Krt9; keratin 9, ko:K07604	–2.73	0.014	0.076
**Multifunctional motif**					
CUST_8465	CNUS0000102763	Cell polarity protein leucine-rich repeat protein scribble complex protein	–2.70	0.001	0.068
CUST_4784	CNUS0000099080	Zinc finger protein 291	–2.04	0.042	0.1

**Figure 6:**
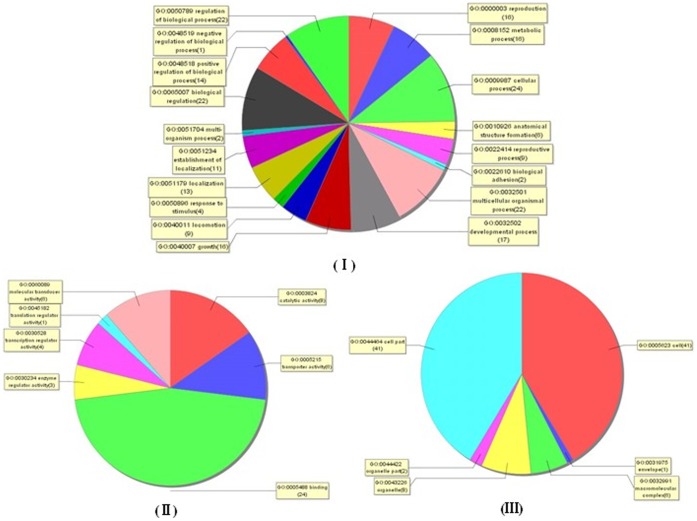
Distribution of gene ontology (GO) terms for the differentially expressed genes in schistosomes from water buffalo and yellow cattle. Pie chart showing the GO classification for biological processes (I), molecular functions (II) and cellular components (III). The chart does not contain those genes that do not have assigned GOs.

A 200-ng aliquot of total RNA from each sample was converted into complementary RNA, labeled with the fluorophore cyanine 3-CTP (CY3c) and hybridized according to the manufacturer’s instructions (Agilent Technologies: One-Color Microarray-Based Gene Expression Analysis). Samples were examined at A260 and A550 using a ND-1000 spectrophotometer (Thermo Scientific, USA) to determine the yield, concentration, amplification efficiency and abundance of CY3c. For the sample from each host, three independent biological replicates were designed for microarray hybridization, which were performed in duplicate for all samples.

**Table 4:** KEGG pathway analysis of differentially expressed genes in schistosomes from water buffalo compared with those from yellow cattle.

Pathway	Class	Molecule in pathway	Orthology/Enzyme	Accession number	FC^a^	Pathway ID
Purine metabolism	Nucleotidemetabolism	Nucleoside diphosphate kinase	K00940/EC:2.7.4.6	CNUS0000102089	2.01	map00230
Pyrimidine metabolism	Nucleotidemetabolism	Nucleoside diphosphate kinase	K00940/EC:2.7.4.6	CNUS0000102089	8.86	map00240
Sphingolipid metabolism	Lipid metabolism	Sphingosine N-acyltransferase	K04710/EC:2.3.1.24	CNUS0000107293	2.01	map00600
Glycosphingolipid biosynthesis -lacto and neolacto series	Glycan biosynthesisand metabolism	N-acetyllactosaminide beta-1,6-N-acetylglucosaminyltransferase	K00742/EC:2.4.1.150	CNUS0000105248	2.96	map00601
Oxidative phosphorylation	Energy metabolism	V-type H+-transporting ATPase subunit H	K02153/EC:3.6.3.14	CNUS0000096235	2.19	map00190
Spliceosome	Transcription	Pre-mRNA-splicing factor SPF27	K12861	FN317168	3.52	map03040
Lysosome	Transport and catabolism	Cathepsin L-like proteinase precursor	K01365/EC:3.4.22.15	CNUS0000097938	2.63	map04142
Wnt signaling pathway	Signal transduction	Protein phosphatase regulatory subunit b alpha isoform	K03456	CNUS0000104279	4.54	map04310
Neuroactive ligand-receptor interaction	Signaling moleculesand interaction	Metabotropic glutamatereceptor 7 precursor	K04608	CNUS0000097512	2.35	map04080

Pathway ID and Enzyme ID of enzymes and orthologies can be searched at http://www.genome.jp/kegg/; Accession number can be searched at http://lifecenter.sgst.cn/schistosoma/cn/genomeProject.do or http://www.ebi.ac.uk/embl/;^ a^Fold changes (FC) are expressed as the ratio of gene expression in schistosomes from water buffalo compared with schistosomes from yellow cattle; *p*<0.05, FDR <0.1.

### Feature Extraction and Data Analysis

Microarrays were scanned using an Agilent Microarray Scanner (G2565BA) at 550 nm. Hybridized slides were scanned as tiff files and processed with the Feature Extraction 9.5.3.1 Image Analysis program (Agilent) to produce standardized data for statistical analysis. All slides were assessed for background evenness by viewing the tiff image using Feature Extraction. Feature-extracted data were analyzed using GENESPRING (version 7.3.1; Agilent Technologies/Silicon Genetics, Redwood City, CA). Microarray data were normalized using a normalization scenario for ‘Agilent FE one-color’, which including ‘Data Transformation: Set measurements less than 5.0 to 5.0′, ‘Per Chip: Normalize to 50th percentile’ and ‘Per Gene: Normalize to median’.

Data sets were further analyzed based on one-colour experiments published previously [17]. The gProcessed Signal values were determined in GENESPRING using Agilent’s Feature Extraction software, including aspects of signal/noise ratio, spot morphology and homogeneity. The gProcessed Signal represents the signal after localized background subtraction and includes corrections for surface trends. Features were deemed ‘Absent’ when the processed signal intensity was less than twice the value of the processed signal error value; ‘Marginal’ when the measured intensity was at a saturated value or if there was a substantial amount of variation in the signal intensity within the pixels of a particular feature. Features that were neither Absent nor Marginal were deemed ‘Present’. Data points were included only if they were Present or Present, Absent and probes or contigs were retained if all data points were Present or Present, Absent. The microarray data were submitted to the GEO public database, under the accession number GSE24615. Raw intensity data were analyzed using the R statistical language [18], [19]. The statistical analysis was performed using the Student’s t test. The q-value estimation for false discovery rate(FDR) control was applied to analyzed the microarray data [20], [21]. Heatmap and PCA were plotted using Java Treeview software (Stanford University, Stanford, CA) and MDS algorithm [22], [23].

### Validation of Microarray Results

A subset of genes predicted to be differentially expressed in microarray analysis was selected for validation using real-time RT-PCR. The gene-specific primers were designed using PRIMER3 (http://frodo.wi.mit.edu/primer3/input.htm). Total amplified RNA (1 µg) from mixed parasites sample from each animal in every group was used for reverse transcription (RT) in a final volume of 20 µL using PrimerScript RT kit with gDNA Eraser (Takara, Cat# DRR047). Products were amplified using the SYBR Premix Ex Taq (Takara, Cat#DRR041A) in an ABI 7500 Realtime System (Applied Biosystems) with the following profile: 50°C for 2 min, 95°C for 30 s; 40 cycles of 95°C for 5 s and 60°C for 34 s; 95°C for 15 s and 60°C for 1 min. Each reaction was performed using 20 µL of cDNA from the RT reaction, to a final volume of 50 µL. Expression levels of *S. japonicum* NADH dehydrogenase (Genebank AY812950) were used as an endogenous control within each sample. From the microarray results, it was noted that the NADH dehydrogenase gene expression does not vary significantly between two hosts. Relative levels of gene expression were calculated using the 2^−ΔΔCT^ method [24]. Each sample was analyzed for primer dimer, contamination or mispriming by advance inspection of their dissociation curves.

### Gene Ontology Analysis

Further analysis was performed at http://www.blast2go.de using Blast2Go Batch BlastX (six frame translation protein homology) [25]. This presented a further overview of gene ontologies (GO) for differentially expressed genes modulated by the two hosts. This software could evaluate the differences in annotation between the data from the two host groups. The analysis of GO terms associated with the genes differentially expressed in schistosomes from the two host groups, was performed by using the combined graphs function of the BLAST software. GO correlations with relative gene expression values were made using ErmineJ software [26]. The KEGG pathway of differentially expressed genes was analyzed using the maps at http://www.genome.jp/kegg [27], [28].

## Results and Discussion

### Comparative Ultrastructure of Schistosomes Derived from Water Buffalo and Yellow Cattle

In addition to the general differences known to occur between hosts, including worm recovery rate, and worm length and width [12], the surface topography and internal structures at the ultrastructural level were also found to differ between schistosomes harvested from water buffalo and yellow cattle. Based on SEM of male schistosome worms derived from water buffalo, the oral sucker was crimpled, tension was attenuated or loosed, the width of the border spine decreased in the ventral sucker, and the surface crest and sensory papillae were flattened in the posterior segment of gynecophoral canal (Fig. 1). Female schistosomes collected from water buffalo showed invagination and collapse of the spines and sensory papillae on the back of the oral sucker, a depressed ventral sucker, a poorly distinguished genital pore, and fewer crest and sensory papillae in middle of the integumentum (Fig. 2). By contrast, female schistosomes from yellow cattle showed relatively normal surface topography, with more spines and sensory papillae in the oral sucker, an evaginated ventral sucker, an obvious genital pore next to the ventral sucker, a compact and regular tegumental ridge and crest and sensory papillae in the middle of the integumentum (Figs 1, 2).

Comparing male worms from yellow cattle with those from water buffalo using TEM showed that those from water buffalo had more vacuolar structures in the tegument, consistent with the collapse and loose surface observed by SEM, and the organelles of cytoplasm had dissolved (Fig. 3); the female worms from water buffalo showed no obvious differences in the tegument compared with worms from yellow cattle, although their internal microvilli were shorter and fewer than in worms from yellow cattle(Fig. 4).

### Global Gene Expression Profiles in Schistosomes from Water Buffalo and Yellow Cattle

Three biological replicates were set for worms from each host, and the correlation of the biological replicates was 0.99 for worms from individual animal of each host. In total, 1484 transcripts (*p*<0.05) were applied for hierarchical clustering. It was possible to identify different profiles from the schistosomes from water buffalo (Group B) and yellow cattle (Group C). The genes of schistosomes from the two different hosts separated into two main clusters (Fig. 5I). To evaluate the overall structure of the data, we plotted the first two principal components using a principal component analysis (PCA). This analysis separated the data into two subgroups, clustering together the biological replicates, and separating the samples by the host from which the schistosomes were derived (Fig. 5II). All the data were deposited in the GEO of NCBI [29], with the GEO Series accession number GSE24615 (http://www.ncbi.nlm.nih.gov/geo/).

For genes to be considered differentially expressed, a two-fold or greater change in gene expression is required, with statistical significance (*p*<0.05 and FDR <0.1). Compared with schistosomes from yellow cattle, microarray analysis revealed that only 69 genes (0.50%) were significant differentially expressed in schistosomes from water buffalo (59 upregulated and 10 downregulated, which are published as Tables S1 and S2 on the PLoS ONE web site). Most of the differentially expressed genes (46/69) in schistosomes from water buffalo versus yellow cattle could also be found in schistosomes from water buffalo and goat, another natural host for *S. japonicum* (data not shown).

### Real-time PCR Validation for Microarray Data

A subset of genes with different expression levels and various biological functions in schistosomes from water buffalo and yellow cattle were selected for real-time RT-PCR to validate the microarray transcription results. The primer sequences and results for validation were listed in Table 1. The RT-PCR results confirmed the results for directionality of regulation, and most of the -fold changes matched the microarray data, thereby validating the results of the microarray.

### GO Functional Distribution Analysis of Differentially Expressed Genes in Schistosomes from Water Buffalo and Yellow Cattle

Most of the differentially expressed genes were mainly involved in biological regulation; metabolic, cellular and developmental processes; growth; reproduction; anatomical structure formation and other biological processes (Fig. 6I ). Molecular function analysis revealed that most of these molecules had binding, catalytic, molecular transducer, transcription regulator and enzyme regulator activities, as well as other important biological functions (Fig. 6II ). Cellular component analysis showed that most of these molecules were involved in the composition of cells, organelles, macromolecular complexes, membrane envelopes and so on (Fig. 6III). The differentially expressed genes were listed in Tables 2 and 3. GO function enrichment analysis and other bioinformatic techniques were further applied to predict and/or analyze the possible function of these genes.

### Comparison of Gene Expression Differences in Schistosomes Derived from Water Buffalo and Yellow Cattle

Gene expression is most abundant and diverse in adult schistosomes because of the need for development, movement, evading host immune attack, the acquisition and metabolism of nutrients, and reproduction. The gene expression difference analysis revealed that, compared with schistosomes from yellow cattle, some genes involved in protein kinase and phosphatase, stimulus response, lipid metabolism, nucleotide metabolism, transcription and translation, were overexpressed in schistosomes from water buffalo, whereas other genes associated with reproduction, anatomical structure morphogenesis and multifunctional motifs were underexpressed. The differential expression of these genes might affect the survival and development of schistosomes in the two natural hosts.

### Analysis of Genes Overexpressed in Schistosomes from Water Buffalo

#### Protein kinase and phosphatase

Protein phosphatase has an important role, alongside protein kinase, in dephosphorylation and/or phosphorylation, which controls a variety of processes, including cell division and growth, signal transduction and metabolism, and influences events ranging from the initiation of DNA replication to vertebrate axis formation to apoptosis. The actions of both pro- and anti-apoptotic factors are often affected by the modulation of the phosphorylation status of key elements of the apoptotic process. Many protein kinases act in apoptosis, of which the serine/threonine protein kinases are known to have a role in apoptosis. Cyclin-dependent kinase (CDK) is a serine/threonine kinase that primes DNA replication and induces mitosis; it also regulates schistosome gene expression associated with embryogenesis [30] and worm paring [31] via the tumor growth factor (TGF)-β signaling pathway; recent studies have also reported a novel role in block differentiation for CDK6 [32], [33]. Downregulation of protein phosphatase 2A (PP2A) activity in the signaling complex can increase growth-promoting β-catenin signaling in the Wnt signaling pathway [34]. Therefore, the overexpression of CDK6 and PP2A revealed by our study in schistosomes from water buffalo would be predicted to block cell differentiation and promote cell apoptosis, resulting in worm clearance and the development of mild pathological damage in water buffalo (Table 2).

#### Stimulus response associated with the nervous system and transport

Schistosomes have a central nervous system and a peripheral sensory nervous system, which help them to attack hosts and reach nutrient-rich areas such as the liver portal vein and intestinal veins [35]. The neural system and its regulation are sensitive to stimulus. Protocadherins are predominantly expressed in the nervous system, and constitute the largest subgroup within the cadherin superfamily of calcium-dependent cell–cell adhesion molecules [36]; glutamate and its receptor are important neurotransmitters in the central nervous system, leading to a complex signal transduction response; Rab3 is a neuronal GTP-binding protein of synaptic vesicles that may function in neurotransmitter release. Other investigators demonstrated that Rab3 is not in itself essential for synaptic membrane traffic but functions to modulate the basic release machinery [37]. The substance exchange between schistosomes and their hosts requires many transporters; therefore, some genes involved in H^+^ transport and nucleoside transport that were also found to be overexpressed in schistosomes from water buffalo, as well as genes involved in neural system regulation, could enable the parasite to adapt to the host environment, enabling them to survive and develop in the host.

#### Genes associated with metabolism

Schistosomes are unable to complete lipid and nucleotide metabolism without their host as they are incapable of the *de novo* synthesis of sterols or free fatty acids and must use complex precursors from the host [38]. The elongase of the very long chain fatty acid (ELOVL) family is responsible for the synthesis of very long chain fatty acids (VLCFAs). The ELOVL family consists of seven members: ELOVL1–7, which are thought to carry out substrate-specific elongation with fatty acids of various lengths and to regulate fatty acid metabolism. In water buffalo, only a few parasites were able to survive and mature. The ELOVL genes were upregulated (CUST_5733 and CUST_10029) in adult schistosomes from water buffalo, perhaps as a result of a compensation mechanism in a less-susceptible host to enable worm growth and development.

Nucleotide metabolism is crucial for schistosomes, but they are unable to synthesize purines by themselves, depending instead on a supply from their host [39]. The current study found that several nucleotide metabolism-associated genes were overexpressed in schistosomes from water buffalo (Table 2). Nucleoside diphosphate kinase (EC 2.7.4.6) is a ubiquitous enzyme that catalyzes phosphorylation of nucleoside 5′-diphosphate, with the exception of ADP, to the corresponding triphosphate. In addition, NDP kinases have been shown to have additional regulatory functions for growth and developmental control, signal transduction, transcription, activation of GTP-binding proteins and tumour metastasis suppression [40]. The structure of NDP kinases is highly conserved from *Escherichia coli* to humans (43% identity) and they are believed to be housekeeping enzymes for DNA and RNA synthesis. However, NDK was upregulated in schistosomes from water buffalo, suggesting that it is also important for worm growth and development in different hosts.

Homeobox genes have fundamental roles in development; in particular, the LIM-homeobox subfamily and Iroquois homeobox family both have transcription factor regulation activity. The primary structure of LIM-homeobox genes has been remarkably conserved through evolution. Previous studies revealed a prominent involvement of LIM-homeodomain proteins in tissue patterning and differentiation, and their function in neural patterning is evident in many organisms [41]. Iroquois homeobox factors are a family of homeodomain transcription factors that have a role in many developmental processes. The *Iro* genes function early in development to specify the identity of diverse territories of the body, such as the dorsal head and dorsal mesothorax of *Drosophila* and the neural plate of *Xenopus* [42]. Pumilio proteins are proposed to have a conserved primordial function in the maintenance of proliferation in stem cells through post-transcriptional regulation. Recent research has also reported that they act as translational repression proteins [43]. Eukaryotic translation initiation factor 3 (eIF3) has a central role in the process of eukaryotic translation initiation. It is a structural center, combined with a variety of eukaryotic initiation factors and RNA to control the entire process of translation initiation.

The genes associated with lipid and nucleotide metabolism, transcription and translation and those were overexpressed in schistosomes from water buffalo, might reflect a response to the requirement for growth and development in the environment of less-susceptible host. However, additional work is required to elucidate more of the details surrounding their involvement in schistosome survival in its natural hosts.

### Analysis of Genes Underexpressed in Schistosomes from Water Buffalo

#### Genes associated with reproduction

ELM2 domain-containing protein is localized to membranes and is associated with calcium ion binding, protein binding and male mating behavior. Members of the importin Beta family are located in the cytoplasm and nucleus, and are associated with embryonic development and reproduction. *Distal-less* homologs, the *Dlx* genes, have roles in reproduction, acting on transcriptional activity during embryonic development. These genes are all involved in reproduction and embryonic development, and all have a positive regulatory role in growth [44] They were found to be underexpressed in schistosomes from water buffalo, which might help explain why schistosomes in water buffalo show restricted growth compared with those in yellow cattle (Table 3).

Long-term host and/or parasite survival depends upon successful modulation of the acute pathological response, which is induced by egg antigens. Cass *et al.* identified egg secretome of *Schistosoma mansoni* and found that it comprised 188 proteins, involved in redox balance, molecular chaperoning and protein folding, development and signaling, scavenging and metabolic pathways, and immune response modulation. After initial infection, egg granuloma formation in the presence of pro-inflammatory cytokines normally results in a shift to a T-helper 2 (Th2)-type anti-inflammatory cytokine environment, directly in response to egg-secreted antigens [45]. The egg-secreted protein (ESP) localized to the integral membrane, is underexpressed in schistosomes sampled from water buffalo, which might be why there was no obvious shift from a Th1 to Th2-type immune response after schistosome infection, as previously reported [12].

Taurine is an amino acid that essential during fetal life and appears to be vital for the growth of the fetus and for the development of the central nervous system. Taurine transporter (TAUT) is responsible for aminoethylsulfonic acid transportation and cell body regulation. In 1998, Staffan *et al.* reported that intrauterine growth restriction is associated with a reduced activity of placental taurine transporters [46].The downregulation of TAUT in schistosomes from water buffalo could explain why schistosomes show poor development and a low survival rate in this host.

#### Genes involved in anatomical structure morphogenesis

The anatomical structure-associated genes, including *Distal-less* homologs (associated with imaginal disc-derived limb morphogenesis and imaginal disc-derived male genitalia development) and ELM2 domain-containing protein (associated with location of a vulva defective), were underexpressed in schistosomes from water buffalo, which was consistent with the structural observations described above.

Schistosome surface membrane molecules have an important role in schistosome–host interactions and are involved in the parasite evasion of immune attack by the host. Membrane molecules, such as transmembrane protein 57, keratin and ESP, were downregulated in schistosomes from water buffalo. Transmembrane proteins, which break through the entire lipid bilayer and are exposed to the external and internal surfaces of the membrane, as well as secreted and transmembrane proteins, are considered to be the main anti-schistosome vaccine or drug targets. Keratin is a type of scleroprotein that has abundant cysteine and disulfide bonds, and is involved in egg-shell protein formation in schistosomes. The scleroproteinaceous schistosome egg shell is lined internally with a vitelline membrane, within which the miracidium develops. The egg shell is a hardened and tanned structure made from crosslinked proteins. It is synthesized within the female worm from many different kinds of protein and glycoprotein and, as with the tegument, could provide a protective biochemical barrier to oxidative stress [47] (Table 3).The underexpression of these genes in schistosomes from water buffalo could be associated with the lower rate of egg production recorded in such worms.

#### Multifunctional motif

The leucine-rich repeat is a recently characterized structural motif used in molecular recognition processes as diverse as signal transduction, cell adhesion, cell development, DNA repair and RNA processing. In 2001, Hugot *et al.* reported that the NOD2 leucine-rich repeat gene product confers susceptibility to Crohn’s disease by overactivating nuclear factor kappa B (NF-kB) in monocytes [48]. Because the adaptive immune molecules (e.g. immunoglobulin) and Toll-like receptors (TLRs) are lacking in schistosomes, the leucine-rich repeat motif was regarded as a potential TLR in schistosomes, possibly with an important role in the innate immune response against *S. japonicum* infection [35]. Therefore, we suggest that the leucine-rich repeat gene downregulated in schistosomes from water buffalo is associated with the innate immune response in that host.

The special finger-like structures, zinc finger proteins, can bind to DNA, protein and RNA, thus having an important role in gene expression, cell differentiation, embryonic development, and so on. The underexpression of zinc finger protein in schistosomes from water buffalo is likely to affect normal biological processes or functions in schistosomes from this less-susceptible host.

### Pathway Analysis of Differentially Expressed Genes in Schistosomes from Water Buffalo and Yellow Cattle

KEGG pathway analysis (Table 4) of differential expressed genes suggested that these genes are involved in many crucial cellular events. The genes were mainly associated with nucleotide metabolism (purine and pyrimidine metabolism), energy metabolism (oxidative phosphorylation), lipid metabolism (sphingolipid metabolism), glycan biosynthesis and metabolism (glycosphingolipid biosynthesis), genetic information processing(transcription), transport and catabolism(lysosome), environmental information processing (Wnt signaling pathway and neuroactive ligand-receptor interaction).

Given that schistosomes are unable to synthesize key nutrient molecules, such as purines, sterols and essential amino acids, they need to use hormones and growth factors from their hosts to support and promote their own growth and development [39], [49]. The differentially expressed genes in schistosomes from two natural hosts examined were mainly associated with nucleotide, lipid,energy metabolism, as well as genetic information processing and environmental information processing, suggesting that the differential expression of some of these genes is associated with the differences seen in the survival and development of schistosomes in different host environments.

### Conclusion

In this study, we compared schistosomes from the *S. japonicum-*susceptible natural host (yellow cattle) with those from the less-susceptible natural host (water buffalo) at both the ultrastructural and transcriptional level. Ultrastructure observations showed that worms from water buffalo have a collapsed and loose surface, with fewer spines and sensory papillae, dissolved cytoplasmic organelles and more vacuole structures in the male worm tegument. The gene expression analysis of the adult worms showed that the differentially expressed genes participated mainly in nucleotide, energy, lipid metabolism, energy metabolism, transcription, transport and signaling pathway and so on. Thus, the differential expression of these genes might affect the survival and development of the schistosome in different natural hosts.

The results from the current study provide a better understanding of the interplay between parasites and their natural hosts, and also valuable information for the screening of schistosome vaccine candidates. If a transmission-blocking veterinary vaccine can be put into practice in combination with other control strategies, such as human chemotherapy, elimination of *S. japonicum* from China, and other endemic areas, might be achievable.

## Supporting Information

Table S1 **Overexpressed genes in schistosomes from water buffalo compared with those from yellow cattle.** The list including the probe name, gene accession, FC (the fold change), regulation and the protein homology(the result of blastx). In the gene accession line, the name such as CNUS0000098407 is from LSBI (http://lifecenter.sgst.cn/schistosoma/cn/genomeProject.do), the name such as FN326902 is from EMBL (http://www.ebi.ac.uk/embl/), the name such as AY809022 is from GeneBank (http://www.ncbi.nlm.nih.gov/genbank/). FDR(*q-value*) is applied for false discovery rate control by R statistical language (http://www.bioconductor.org/packages/release/bioc/html/qvalue.html).
(DOC)

Table S2 **Underexpressed genes in schistosomes from water buffalo compared with those from yellow cattle.**
(DOC)
