# Supplementary material for: Ultrastructural Observation and Gene Expression Profiling of Schistosoma japonicum Derived from Two Natural Reservoir Hosts, Water Buffalo and Yellow Cattle
Source: PLoS One. 2012 Oct 26;7(10):e47660. doi: 10.1371/journal.pone.0047660 (PMC3482235; doi:10.1371/journal.pone.0047660)
Supplement: Table S1 — Overexpressed genes in schistosomes from water buffalo compared with those from yellow cattle. The list including the probe name, gene accession, FC (the fold change), regulation and the protein homology(the result of blastx). In the gene accession line, the name such as CNUS0000098407 is from LSBI (http://lifecenter.sgst.cn/schistosoma/cn/genomeProject.do), the name such as FN326902 is from EMBL (http://www.ebi.ac.uk/embl/), the name such as AY809022 is from GeneBank (http://www.ncbi.nlm.nih.gov/genbank/). FDR(q-value) is applied for false discovery rate control by R statistical language (http://www.bioconductor.org/packages/release/bioc/html/qvalue.html). (DOC) [file pone.0047660.s001.doc]

**Tables S1 Over-expressed genes in schistosomes from water buffalo compared to those from yellow cattle.**

| ***Probe name*** | ***Accession number*** | ***Gene*** | ***FC*** | ***p value*** | ***FDR*** | ***Protein Homology*** |
| --- | --- | --- | --- | --- | --- | --- |
| CUST_4112 | CNUS0000098407 | Cyclin-dependent kinase 6 | 18.52 | 0.029 | 0.092 | Cyclin-dependent kinase 6 |
| CUST_12988 | CNUS0000107293 | Putative uncharacterized protein C14orf165 | 8.86 | 0.007 | 0.073 | Uncharacterized protein c14orf165-like |
| CUST_10057 | CNUS0000104355 | Protocadherin gamma B2 precursor | 7.95 | 0.036 | 0.092 | Protocadherin alpha subfamily 2 |
| CUST_10350 | CNUS0000104648 | Asparagine-rich protein | 6.11 | 0.003 | 0.084 | --NA--- |
| CUST_1782 | CNUS0000096077 | Eukaryotic translation initiation factor 3 subunit 12 | 5.33 | 0.009 | 0.092 | Eukaryotic translation initiation factor 3 subunit k |
| CUST_9981 | CNUS0000104279 | Serine/threonine-protein phosphatase 2A | 4.54 | 6.51E-05 | 0 | Protein phosphatase regulatory subunit b alpha isoform,PP2A |
| CUST_7279 | CNUS0000101576 | Expressed protein | 4.51 | 0.002 | 0.084 | --NA--- |
| CUST_10723 | CNUS0000105021 | Expressed protein | 4.21 | 0.036 | 0.092 | sjchgc03548 protein |
| CUST_2857 | CNUS0000097152 | IPR008952 Tetraspani | 4.2 | 0.015 | 0.092 | --NA--- |
| CUST_12961 | CNUS0000107266 | Expressed protein, putative, mRNA | 3.81 | 0.007 | 0.092 | --NA--- |
| CUST_191 | FN320911 | Hypothetical protein | 3.6 | 0.039 | 0.095 | --NA--- |
| CUST_488 | FN317168 | Hypothetical protein | 3.52 | 0.015 | 0.092 | sjchgc08923 protein， T12A2.7 hypothetical protein |
| CUST_1819 | CNUS0000096114 | Hypothetical protein | 3.39 | 0.017 | 0.092 | ---NA--- |
| CUST_8346 | CNUS0000102644 | Leukocyte surface antigen CD53 (Cell surface glycoprotein CD53) | 3.01 | 0.049 | 0.095 | ---NA--- |
| CUST_10950 | CNUS0000105248 | Beta-1,3-galactosyl-O-glycosyl-glycoprotein beta-1,6-N-acetylglucosaminyltransferase (EC 2.4.1.102) (Core 2 branching enzyme) (Core2-GlcNAc-transferase) (C2GNT) (Core 2 GNT) | 2.96 | 0.036 | 0.095 | ---NA--- |
| CUST_102 | lcl|T1-2H06 | - | 2.85 | 0.024 | 0.092 | ---NA--- |
| CUST_4101 | CNUS0000098396 | Iroquois-class homeodomain protein IRX-3 (Iroquois homeobox protein 3) (Homeodomain protein IRXB1) | 2.66 | 0.031 | 0.092 | Iroquois homeobox family transcription factor |
| CUST_277 | FN321589 | Hypothetical protein | 2.64 | 0.011 | 0.092 | ---NA--- |
| CUST_3643 | CNUS0000097938 | Cathepsin L-like proteinase precursor (EC 3.4.22.-) | 2.63 | 0.005 | 0.092 | ---NA--- |
| CUST_6369 | CNUS0000100665 | Hypothetical protein | 2.61 | 0.011 | 0.092 | ---NA--- |
| CUST_6737 | CNUS0000101033 | Hypothetical protein | 2.58 | 0.039 | 0.095 | Hypothetical protein [S. japonicum] |
| CUST_5733 | CNUS0000100029 | Elongation of very long chain fatty acids protein 1 | 2.57 | 0.039 | 0.092 | Elongation of very long chain fatty acids (fen1 sur4 yeast)-like 1 |
| CUST_1079 | AY809022 | SJCHGC02234 protein mRNA, partial cds | 2.56 | 0.031 | 0.092 | sjchgc02234 protein |
| CUST_13686 | CNUS0000107991 | Conserved hypothetical protein, IPR000477 RNA-directed DNA polymerase (Reverse transcriptase) | 2.45 | 0.014 | 0.092 | ---NA--- |
| CUST_8181 | CNUS0000102479 | Hypothetical protein | 2.42 | 0.039 | 0.095 | Hypothetical protein [S. japonicum] |
| CUST_694 | FN318567 | Hypothetical protein | 2.4 | 0.029 | 0.095 | Hypotheticial protein |
| CUST_485 | FN317134 | Hypothetical protein | 2.39 | 0.019 | 0.092 | ---NA--- |
| CUST_12656 | CNUS0000106958 | Hypothetical protein | 2.37 | 0.034 | 0.095 | C50F2.3 hypothetical protein |
| CUST_11457 | CNUS0000105756 | Histone H4 | 2.36 | 0.006 | 0.092 | Histone h4-like |
| CUST_3217 | CNUS0000097512 | Metabotropic glutamate receptor 7 precursor (mGluR7) | 2.35 | 0.04 | 0.092 | ---NA--- |
| CUST_11472 | CNUS0000105771 | Hypothetical protein | 2.34 | 0.002 | 0.084 | Hypothetical protein [S.mansoni] |
| CUST_2605 | CNUS0000096900 | Histone H4 | 2.272509 | 0.009 | 0.092 | Histone h4-like |
| CUST_1041 | FN326681 | Hypothetical protein | 2.25 | 0.022 | 0.092 | Hypotheticial protein |
| CUST_2965 | CNUS0000097260 | LIM/homeobox protein Lhx1 (LIM homeobox protein 1) | 2.23 | 0.024 | 0.092 | LIM/homeobox transcription factor alpha |
| CUST_8562 | CNUS0000102860 | Protein VHS3 (Viable in a HAL3 SIT4 background protein 3) | 2.21 | 0.026 | 0.092 | ---NA--- |
| CUST_88 | lcl|f4-c06-t7p | - | 2.2 | 0.015 | 0.092 | ---NA--- |
| CUST_738 | FN318954 | Hypothetical protein | 2.2 | 0.034 | 0.092 | Hypotheticial protein |
| CUST_8688 | CNUS0000102986 | Expressed protein | 2.19 | 0.028 | 0.092 | sjchgc08962 protein |
| CUST_1940 | CNUS0000096235 | Hypothetical protein MGC81224 [EC:3.6.3.14]; ko:K02153 V-type H+-transporting ATPase subunit H | 2.19 | 0.04 | 0.095 | v-type proton ATPase subunit e 1 |
| CUST_4696 | CNUS0000098991 | Hypothetical protein MGC68579 [EC:2.7.11.2]; ko:K00898 pyruvate dehydrogenase kinase | 2.19 | 0.049 | 0.092 | ---NA--- |
| CUST_4513 | CNUS0000098808 | Hypothetical protein | 2.18 | 0.015 | 0.092 | ---NA--- |
| CUST_11483 | CNUS0000105782 | Ecotropic virus integration site 1 protein (EVI-1) | 2.12 | 0.012 | 0.092 | mds1 and evi1 complex locus protein evi1 |
| CUST_739 | FN318955 | Hypothetical protein | 2.12 | 0.033 | 0.092 | Hypotheticial protein |
| CUST_8420 | CNUS0000102718 | Hypothetical protein | 2.11 | 0.04 | 0.092 | Pumilio [S.mansoni] |
| CUST_6314 | CNUS0000100610 | Expressed protein | 2.1 | 0.038 | 0.095 | ---NA--- |
| CUST_12911 | CNUS0000107215 | Derlin-2 (Degradation in endoplasmic reticulum protein 2) (Der1-like protein 2) | 2.1 | 0.038 | 0.095 | ---NA--- |
| CUST_13750 | lcl|chgc_new_contig1418 | - | 2.1 | 0.002 | 0.084 | ---NA--- |
| CUST_7137 | CNUS0000101434 | 39S ribosomal protein L35, mitochondrial precursor (L35mt) | 2.08 | 0.01 | 0.092 | 39s ribosomal protein mitochondrial |
| CUST_49 | lcl|chgc_new_contig1630 | - | 2.07 | 0.024 | 0.092 | ---NA--- |
| CUST_12650 | CNUS0000106952 | Conserved hypothetical protein | 2.07 | 0.022 | 0.092 | Lyr motif-containing protein 1 |
| CUST_2092 | CNUS0000096387 | Regulating synaptic membrane exocytosis protein 2 (Rab3-interacting molecule 2) (RIM 2) (Rab3-interacting protein 2) | 2.07 | 0.018 | 0.092 | Rab3 interacting molecule -related |
| CUST_13757 | lcl|f06-d04 | - | 2.07 | 0.016 | 0.092 | ---NA--- |
| CUST_125 | FN313838 | Hypothetical protein | 2.05 | 0.018 | 0.092 | ---NA--- |
| CUST_13650 | CNUS0000107955 | Retrovirus-related Pol polyprotein from transposon 17.6 [Includes: Protease (EC 3.4.23.-); Reverse transcriptase (EC 2.7.7.49); Endonuclease] | 2.05 | 0.033 | 0.092 | ---NA--- |
| CUST_4690 | CNUS0000098985 | Hypothetical protein | 2.04 | 0.023 | 0.092 | ---NA--- |
| CUST_11279 | CNUS0000105578 | Hypothetical protein | 2.04 | 0.004 | 0.092 | ---NA--- |
| CUST_5523 | CNUS0000099819 | Putative eukaryotic translation initiation factor 3 subunit (eIF-3) | 2.03 | 0.037 | 0.092 | Eukaryotic translation initiation factor 3 subunit (eif-3) |
| CUST_7791 | CNUS0000102089 | LOC476638; similar to nucleoside diphosphate kinase type 6; ko:K00940 nucleoside-diphosphate kinase | 2.02 | 0.027 | 0.092 | Nucleoside diphosphate kinase 6 |
| CUST_12289 | CNUS0000106588 | Hypothetical protein | 2.01 | 0.035 | 0.092 | Equilibrative Nucleoside Transporter， ent-1 |
