# Supplementary material for: Ultrastructural Observation and Gene Expression Profiling of Schistosoma japonicum Derived from Two Natural Reservoir Hosts, Water Buffalo and Yellow Cattle
Source: PLoS One. 2012 Oct 26;7(10):e47660. doi: 10.1371/journal.pone.0047660 (PMC3482235; doi:10.1371/journal.pone.0047660)
Supplement: Table S2 — Underexpressed genes in schistosomes from water buffalo compared with those from yellow cattle. (DOC) [file pone.0047660.s002.doc]

Tables S2 Under-expressed genes in schistosomes from water buffalo compared to those from yellow cattle.

| ***Probe name*** | ***Accession number*** | ***Gene*** | ***FC*** | ***p value*** | ***FDR*** | ***Protein Homology*** |
| --- | --- | --- | --- | --- | --- | --- |
| CUST_882 | FN326902 | Hypothetical protein | -20.47 | 0.02 | 0.068 | Putative Transmembrane protein 57 [Schistosoma japonicum] |
| CUST_862 | FN326855 | Hypothetical protein | -16.1 | 0.042 | 0.068 | Egg secreted protein ESP15-like [Schistosoma mansoni] |
| CUST_10799 | CNUS0000105097 | Conserved hypothetical SJCHGC09538 protein | -2.92 | 0.024 | 0.068 | imb-3 IMportin Beta family |
| CUST_12613 | CNUS0000106914 | Hypothetical protein, | -2.92 | 0.022 | 0.086 | sjchgc02128 protein |
| CUST_8162 | CNUS0000102460 | Krt9; keratin 9; ko:K07604 type I keratin | -2.73 | 0.014 | 0.076 | Krt9; keratin 9 [S. japonicum] |
| CUST_8465 | CNUS0000102763 | Expressed protei | -2.7 | 0.001 | 0.068 | Cell polarity protein leucine-rich repeat protein scribble complex protein |
| CUST_11761 | CNUS0000106060 | Hypothetical protein [EC:2.3.2.8]; ko:K00685 arginine-tRNA-protein transferase | -2.22 | 0.043 | 0.100 | Homeobox protein distal-less dlx |
| CUST_13255 | CNUS0000107560 | IPR000949 ELM2,domain-containing protein | -2.08 | 0.028 | 0.095 | Hypothetical protein [Schistosoma mansoni] |
| CUST_4819 | CNUS0000099115 | SLC6A12; solute carrier family 6 (neurotransmitter transporter), member 12; ko:K05045 | -2.07 | 0.006 | 0.068 | Taurine transporter, neurotransmitter transporter |
| CUST_4784 | CNUS0000099080 | Zinc finger protein 291 | -2.04 | 0.042 | 0.100 | Hypothetical protein [Schistosoma mansoni]；S phase cyclin A-associated protein in the endoplasmic reticulum |
